# Biotransformation and bioaccessibility of active ingredients from *Radix Astragali* by *Poria cocos* during solid-state fermentation and in vitro digestion and antioxidant activity evaluation

**DOI:** 10.1038/s41598-023-33969-4

**Published:** 2023-04-27

**Authors:** Cai-Yun Chen, Run Zhang, Li-Jie Zhang, Zhi-Yong Hu, Shao-Ping Wang, Xue Mei, Wei Mi, Jia-Yu Zhang

**Affiliations:** 1 School of Public Health and Management, Binzhou Medical University, Yantai, People’s Republic of China; 2 School of Pharmaceutical Science, Binzhou Medical University, Yantai, 264003 People’s Republic of China

**Keywords:** Medicinal chemistry, Pharmaceutics, Microbiology, Applied microbiology

## Abstract

Radix Astragali is one of the most famous and frequently used health food supplements and herbal medicines. Among more than 227 components of *Radix Astragali*, Astragaloside IV (AG IV) is famous functional compound and is commonly used as a quality marker for *Radix Astragali*. However, the relatively low content of AG IV in *Radix Astragali* (< 0.04%, w/w) severely limits its application. The purpose of this study is to improve the biotransformation of AG IV and its bioaccessibility during in vitro digestion by *Poria cocos* solid fermenting *Radix Astragali*. The optimum fermentation conditions were as follows: Inoculation amount 8 mL; fermentation time 10 d; fermentation humidity 90%. Through fermentation, the content of AG IV was increased from 384.73 to 1986.49 μg/g by 5.16-fold. After in vitro digestion, the contents of genistin, calycosin, formononetin, AG IV, Astragaloside II (AG II) and total flavonoids in fermented *Radix Astragali* (FRA) of enteric phase II (ENTII) were 34.52 μg/g, 207.32 μg/g, 56.76 μg/g, 2331.46 μg/g, 788.31 μg/g, 3.37 mg/g, which were 2.08-fold, 2.51-fold, 1.05-fold, 8.62-fold, 3.22-fold and 1.50-fold higher than those of control, respectively. The Scanning electron microscopy (SEM) of FRA showed rough surface and porous structure. The DPPH and ABTS radical scavenging rate of FRA were higher than those of control. These results showed that the *Poria cocos* solid fermentation could increase the content of the AG IV in *Radix Astragali* and improve the bioaccessibility and antioxidant activity of *Radix Astragali*, which is providing new ideas for future development and utilization of *Radix Astragali*.

## Introduction

*Radix Astragali* is the dried root of *Astragalus membranaceus* (Fisch.) Bge. var. *mongholicus* (Bge.) Hsiao or *A. membranaceus* (Fisch.) Bge. in the leguminous family. It is a famous traditional medicine and healthy food supplement in China with the beneficial effects for human health^1^. Furthermore, it is widely applied as a health additive in foods, teas, drinks, wines and so on. *Radix Astragali* is abundant with biologically active compounds, such as astragalosides, flavonoids, polysaccharide and various trace elements^2^. Among them, AG IV is the most significant compound for the pharmacological activities and therapeutic efficacy. Modern pharmacological studies showed that it has good effects on the cardiovascular system, immune, digestive, nervous system. There were many therapeutic effects for AG IV, such as anti-inflammatory, anti-fibrotic, anti-oxidative stress, anti-asthma, anti-diabetes, immunoregulation and cardioprotective effect^3,4^. However, the extraction rate of AG IV in *Radix Astragali* is limited by the conventional production technology. And there are lots of lignin, cellulose and pectin tightly bound to bioactive compounds in *Radix Astragali* and these substances inhibit the release of bioactive compounds into the digestive system. Thus, in order to solve this problem, some studies are devoted to microbe ferment *Radix Astragali*. The microbial fermentation can produce multienzyme system, which can transform the other astragalosides to AG IV^5^. On the other hand, the enzymes can hydrolyze the lignin, cellulose and pectin of *Radix Astragali*^6^, which can promote the release of the AG IV.

*Poria cocos* is the dried sclerotium of *Poria cocos* (Schw.)Wolf., which is an important edible and medicinal fungus. It has a long history of medicinal use in China and other Asian countries^7^. Research has demonstrated that *Poria cocos* have biological activities described as adjusting the immune function, antitumor and anti-inflammatory, etc^8^. In recent decades, the fermentation culture of *Poria cocos* has been developed because of the potential for the increased production of mycelia and bioactive components in a compact space and in a shorter period. During the fermentation process, *Poria cocos* makes full use of cellulose and other components as a carbon source to degrade the cell wall of the plant, which is contribute to the release of the bioactive compounds of the plant. In addition, as a consequence of its nutritional and health values, it is also applied as functional food. The combined use of *Radix Astragali* and *Poria cocos* has a long history, with the effect of tonifying the spleen and kidney. So far, there are no reports about fermentation of *Radix Astragali* using *Poria cocos*.

Currently, in vitro digestion techniques are commonly used to study the bioaccessibility of herbs and foods in order to better investigate the physicochemical changes and metabolism of functionally active substances during digestion. The microbial fermentation can promote the release of bioactive compounds into the digestive system. In order to investigate the bioaccessibility and digestion of *Radix Astragali* before and after fermentation, in vitro digestion experiments were conducted in the study. In vitro digestion system is an in vitro model for predicting or evaluating the digestibility, bioaccessibility, release kinetic characteristics and structural changes of compounds based on human gastrointestinal tract physiological processes and simulating in vivo digestion and absorption under in vitro conditions. The system can completely or partially replace in vivo tests and has the advantages of cost savings, shorter test cycles, accuracy and manual monitoring.

In this study, *Radix Astragali* was fermented by *Poria cocos* and used in vitro digestion and antioxidant technique to investigate the bioaccessibility and antioxidant activity before and after fungus fermentation, which provided a new idea for the future research of *Radix Astragali*.

## Results and discussion

### Dynamic growth curve of Poria cocos

**Figure 1:**
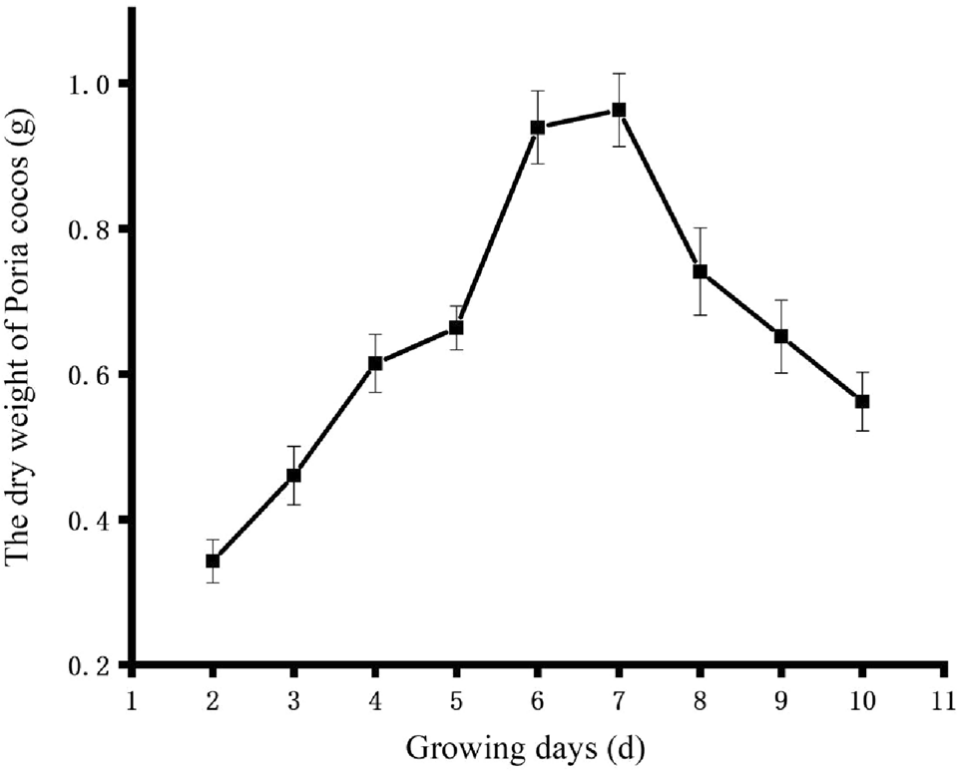
Growth curves of *Poria cocos.*

A time-course study was performed on mass accumulation of *Poria cocos* cultured in 100 mL of 26.1 g/L potato liquid medium. The kinetics of the growth curves of *Poria cocos* was determined initially (Fig. 1). From 2 to 6 days, the dry weight of mycelium increased rapidly, which is the logarithmic phase of *Poria cocos.* On the 6th day, the growth trend of *Poria cocos* tends to stabilize. The peaks occurred at 7 d . Beyond 7 d, the culture entered a senescence phase. At the 7th day, the culture achieved the maximal mass accumulation and the production of secondary metabolites reached its maximum. According to the growth curve of *Poria cocos* to determine the incubation time of *Poria cocos*^9^.

### Optimisation of fermentation procedure

Flavonoids and saponins are the basic active ingredients of plant extracts. The contents of flavonoids and saponins compounds present in fermentation *Radix Astragali* extract was determined by spectrophotometer and HPLC. After fermentation, the contents of AG IV and genistein increased. The factors concerning *Poria cocos* solid fermented *Radix Astragali* include inoculation amount, fermentation time and fermentation humidity. The influence of each factor was studied by single-factor experiments. All assays were conducted in triplicate.

### Effect of inoculation amount

The effect of inoculation amount on fermentation was studied under the conditions of fermentation temperature of 27 °C, fermentation time of 7 d, fermentation humidity of 90%. The inoculation amount ranged from 4 to 9 mL for the single-factor optimization test (Table 1). After fermentation, the contents of flavonoids, saponins and other active components in *Radix Astragali* showed different trends. The contents of calycosin-7-*O*-β-d-glucopyranoside (calycosin-glu), ononin, calycosin and formononetin were reduced significantly. The content of total saponins changed little and the content of total flavonoids increased. As the inoculation amount increased from 4 to 8 mL, the contents of genistein and AG IV increased. When the inoculation amount exceeded 8 mL, the contents of both compounds dereased. The reduction in the contents of calycosin-glu and other components may be due to hydrolytic enzymes produced by *Poria cocos*. These components were further converted to other secondary metabolite or smaller molecules^10^. The *Poria coco*s in growth and reproduction process may produce some enzymes, which can degrade cellulose, pectin and other substances in the cell wall of *Radix Astragali* and facilitate the release of chemical components. The increase of AG IV content may be due to the metabolism or biotransformation of other astragalosides as precursors in the fermentation process^11^. With the increase of inoculation amount, the contents of AG IV and other components decreased. Therefore, 8 mL of seed fermentation broth was chosen as the optimal inoculation amount.

**Table 1:** Contents of bioactive compounds in inoculation amount.

Component	Inoculation amount (mL)						
	Control	4	5	6	7	8	9
Calycosin-glu	287.36 ± 0.60^a^	262.65 ± 0.97^b^	192.38 ± 1.07^c^	160.18 ± 1.27^d^	149.78 ± 1.59^e^	51.00 ± 0.51^g^	72.49 ± 0.48^f^
Genistin	9.53 ± 0.27^e^	12.50 ± 0.93^d^	12.75 ± 0.75^d^	15.19 ± 0.79^c^	16.20 ± 0.58^c^	25.15 ± 0.39^a^	20.86 ± 0.69^b^
Ononin	124.44 ± 0.46^a^	112.36 ± 1.10^b^	89.79 ± 0.68^c^	75.38 ± 1.16^d^	70.14 ± 1.31^e^	23.49 ± 0.61^ g^	37.54 ± 0.76^f^
Calycosin	106.66 ± 0.45^a^	85.36 ± 1.16^b^	71.29 ± 0.40^c^	62.93 ± 0.43^d^	52.52 ± 0.91^e^	43.91 ± 0.31^ g^	47.75 ± 0.48^f^
Formononetin	71.33 ± 0.97^a^	48.85 ± 0.47^b^	39.30 ± 0.85^c^	29.83 ± 0.68^d^	22.48 ± 0.31^e^	11.75 ± 0.29f.	13.52 ± 0.31^g^
AG IV	80.48 ± 0.63^g^	107.94 ± 0.71f.	110.43 ± 1.03^e^	120.65 ± 0.82^d^	223.27 ± 1.43^b^	270.82 ± 0.49^a^	143.76 ± 1.32^c^
AG II	150.01 ± 0.63^d^	151.57 ± 1.10^c^	121.56 ± 0.91f.	147.87 ± 0.13^e^	261.49 ± 0.99^a^	158.19 ± 0.34^b^	158.3 ± 0.30^b^
Total saponins	46.46 ± 0.63^b^	54.11 ± 0.35^a^	39.42 ± 0.88^c^	54.76 ± 0.36^a^	36.02 ± 0.88^d^	35.13 ± 0.25^d^	31.37 ± 0.99^e^
Total flavonoids	1.48 ± 0.09^c^	2.16 ± 0.08^b^	2.18 ± 0.12^b^	2.54 ± 0.08^a^	2.51 ± 0.27^a^	2.65 ± 0.13^a^	2.72 ± 0.12^a^

Values are expressed as mean ± standard error from three replications. Means with different superscripts in the same row show significant difference (P < 0.05). Unit of calycosin-glu, genistin, ononin, calycosin, formononetin, AG IV and AG II here is μg/g. Unit of total saponins and total flavonoids here is mg/g.

### Effect of fermentation time

The effect of fermentation time was studied under the conditions of fermentation temperature of 27 °C, inoculation amount of 8 mL, fermentation humidity of 90%. The fermentation time ranged from 6 d-11 d for the single-factor optimization experiment (Table 2). The changes of the contents of flavonoids, saponins and other active ingredients in *Radix Astragali* after fermentation were fluctuated. The contents of calycosin-glu, ononin, calycosin, formononetin, and AG II were significantly reduced after fermentation. The content of total saponins was lower than that of control and the content of total flavonoids increased. With the increase of fermentation days, the contents of genistin and AG IV increased first and then decreased. The maximum content of genistin was 34.75 μg/g on the 7th day and the maximum content of AG IV was 775.79 μg/g on the 10th day of fermentation. *Poria cocos* grew and metabolized vigorously in the early stage of fermentation. However, with the increase of fermentation days, the strain gradually aging, nutrient consumption increased, and metabolites reduced^12^. At 10 days, the mass accumulation of the content of AG IV achieved the maximum, so 10 days was chosen as the optimal fermentation time.

**Table 2:** Contents of bioactive compounds in different fermentation time.

Component	Fermentation time(d)						
	Control	6	7	8	9	10	11
Calycosin-glu	60.50 ± 1.09^a^	13.32 ± 0.28^c^	16.42 ± 0.52^b^	16.85 ± 0.54^b^	15.83 ± 0.28^b^	16.83 ± 0.31^b^	15.90 ± 0.95^b^
Genistin	22.01 ± 0.50^d^	22.14 ± 0.05^d^	34.75 ± 0.87^a^	30.54 ± 1.15^b^	22.61 ± 0.89^d^	28.72 ± 0.74^c^	28.51 ± 0.88^c^
Ononin	29.83 ± 0.65^a^	19.47 ± 0.49^cd^	20.62 ± 0.28^b^	19.74 ± 0.12^c^	16.26 ± 0.15f.	18.73 ± 0.43^e^	18.82 ± 0.26^de^
Calycosin	282.80 ± 1.79^a^	141.72 ± 0.32^e^	130.51 ± 0.93^g^	177.37 ± 0.92^c^	139.16 ± 1.56f.	156.10 ± 0.69^d^	220.03 ± 1.88^b^
Formononetin	133.07 ± 2.64^a^	51.07 ± 0.10^b^	43.90 ± 0.88^c^	43.53 ± 0.45^c^	43.27 ± 0.79^c^	39.58 ± 1.00^d^	51.60 ± 1.10^b^
AG IV	298.06 ± 2.15^f^	485.64 ± 0.61^d^	478.88 ± 0.83^e^	552.16 ± 1.92^b^	486.41 ± 1.22^d^	775.79 ± 0.62^a^	537.02 ± 1.51^c^
AG II	436.16 ± 1.24^a^	338.45 ± 0.80^c^	362.42 ± 1.34^b^	242.11 ± 0.37^e^	302.61 ± 0.90^d^	166.64 ± 0.96^ g^	187.51 ± 0.69^f^
Total saponins	54.38 ± 0.81^a^	32.34 ± 1.03^de^	34.11 ± 0.16^c^	31.26 ± 0.68^e^	33.77 ± 1.10^ cd^	37.72 ± 1.03^b^	33.46 ± 0.90^cd^
Total flavonoids	3.08 ± 0.21^d^	4.48 ± 0.25^ab^	4.61 ± 0.11^ab^	4.44 ± 0.25^b^	3.81 ± 0.23^c^	4.93 ± 0.21^a^	4.92 ± 0.38^a^

Values are expressed as mean ± standard error from three replications. Means with different superscripts in the same row show significant difference (P < 0.05). Unit of calycosin-glu, genistin, ononin, calycosin, formononetin, AG IV and AG II here is μg/g. Unit of total saponins and total flavonoids here is mg/g.

### Effect of fermentation humidity

The fermentation humidity was investigated using different humidity-e.g., 65%, 90%, 115%, 140% and 165%-under the conditions of fermentation temperature of 27 °C, inoculation amount of 8 mL, fermentation time of 10 d (Table 3). With the increase of humidity, the contents of AG IV, AG II and total flavonoids increased and then decreased, and the content of genistin increased significantly. The highest content of genistein was 48.65 μg/g at the humidity of 165% and the highest content of AG IV was 1986.49 μg/g at the humidity of 90%. The Table 3 showed that with the increase of humidity from 65 to 90%, the content of AG IV increased gradually. However, with the increase of humidity to 165%, the content of AG IV reduced. Maybe the increase in fermentation humidity reduced the density of strains, released the contact inhibition of *Poria cocos*, which helped *Poria cocos* growth and metabolism. However, too much water caused the residue of *Radix Astragali* to produce stickiness, which produced a poor permeability and less dissolved oxygen environment for *Poria cocos*. It was not conducive to the growth and metabolism of *Poria cocos*. Because the content of AG IV increased the most when the humidity was 90%, 90% was chosen as the optimal fermentation humidity.

**Table 3:** Contents of bioactive compounds in humidity.

Component	Humidity (%)					
	Control	65	90	115	140	165
Calycosin-glu	433.09 ± 2.35^a^	211.75 ± 1.43^b^	155.51 ± 1.50^c^	156.65 ± 0.51^c^	141.99 ± 1.70^d^	110.66 ± 1.05^e^
Genistin	10.00 ± 0.65^f^	23.31 ± 0.89^e^	37.44 ± 0.89^c^	34.58 ± 0.86^d^	39.69 ± 0.84^b^	48.65 ± 0.87^a^
Ononin	144.63 ± 1.62^a^	65.42 ± 0.92^b^	54.85 ± 0.89^c^	36.91 ± 0.66^f^	48.94 ± 0.27^d^	46.41 ± 0.49^e^
Calycosin	155.05 ± 0.92^a^	88.39 ± 0.90^e^	75.57 ± 1.32^f^	93.80 ± 1.55^d^	98.49 ± 0.91^c^	110.62 ± 1.21^b^
Formononetin	59.89 ± 0.57^a^	29.74 ± 0.15^b^	21.78 ± 0.62^c^	22.36 ± 0.84^c^	22.63 ± 0.35^c^	18.82 ± 0.88^d^
AG IV	384.73 ± 2.53^f^	701.02 ± 2.39^e^	1986.49 ± 1.83^a^	1144.78 ± 1.72^d^	1310.80 ± 0.85^c^	1545.76 ± 2.24^b^
AG II	540.99 ± 1.30^d^	466.52 ± 0.93^f^	942.61 ± 1.27^a^	553.25 ± 0.95^b^	489.76 ± 1.25^e^	544.36 ± 1.18^c^
Total saponins	56.98 ± 0.88^a^	45.49 ± 1.09^c^	31.09 ± 0.65^e^	49.53 ± 1.10^b^	41.18 ± 0.82^d^	49.85 ± 0.81^b^
Total flavonoids	2.32 ± 0.28^c^	4.41 ± 0.17^ab^	2.69 ± 0.12^c^	4.55 ± 0.38^a^	4.05 ± 0.12^b^	4.49 ± 0.31^ab^

Values are expressed as mean ± standard error from three replications. Means with different superscripts in the same row show significant difference (P < 0.05). Unit of calycosin-glu, genistin, ononin, calycosin, formononetin, AG IV and AG II here is μg/g. Unit of total saponins and total flavonoids here is mg/g.

In the single-factor fermentation experiments, the changes of the contents of flavonoids, saponins and other active ingredients were fluctuated. Combined with the growth curves of *Poria cocos* (Fig. 1), the changes of the contents may be due to the growth of *Poria cocos*, which influnced the biotransformation of *Radix Astragali*. The fermentation conditions caused great fluctuations in the content of AG II. This phenomenon may be caused by the biotransformation of saponins, the other saponins first converted to AG II and then converted to AG IV^13^.

A thorough analysis of the tabular data showed the content of AG IV of the maximum factor for the optimal conditions. Meanwhile, AG IV is officially used as a quality-marker for *Radix Astragali* in Chinese Pharmacopoeia. The optimum fermentation conditions of the fermentation were as follows: 20 g of *Radix Astragali*, 90% humidity and 8 mL of inoculation amount were added and fermented at 27 °C for 10 days. In the optimum fermentation conditions, the content of AG IV was 1986.49 μg/g, which was 5.16 times higher than the control.

### Bioconversion and bioaccessibility of the main active ingredients of Radix Astragali during in vitro digestion process

Bioaccessibility can be defined as the quantity which is released from the food matrix in the gastrointestinal tract and becomes available for absorption. However, it is difficult to study the in vivo changes of these components during their passage through the digestive tract. The in vitro measurement of bioaccessibility is now considered to be a reliable way to assess the availability of dietary compounds. The in vitro simulated gastrointestinal method consists of four phases: oral phase (OP), gastric phase (GP), enteric phase I (ENTI) and ENTII.

Table 4 shows the effects of in vitro digestion on the contents changes of main effective components in *Radix Astragali* before and after fermentation. The contents of genistin, calycosin, formononetin, AG IV, AG II in the FRA increased significantly, and the contents of each component in ENTII were 34.52, 207.32, 56.76, 2331.46 and 788.31 μg/g, which were 2.08-fold, 2.51-fold, 1.05-fold, 8.62-fold and 3.22-fold higher than those of the control, respectively. This is due to solid-state fermentation led to the destruction of the cell wall of *Radix Astragali*, promoting the release of flavonoids and saponins. On the other hand, gastrointestinal digestion significantly enhanced the release of effective components of *Radix Astragali*. Due to the action of the gastrointestinal digestive enzymes, the cellular structure of *Radix Astragali* and chemical bond of acetyl group and glycosidic were destroyed, and combined flavonoids and saponins were released^14^. The contents of calycosin glucoside and ononin decreased significantly.

**Table 4:** Compounds contents of FRA and control during different in vitro digestion stages and bioaccessibility.

Component	Oral phase (OP)		Gastric phase (GP)		Enteric phase I (ENTI)		Enteric phase II (ENTII)		The bioaccessibility (%)	
	Control	After fermentation	Control	After fermentation	Control	After fermentation	Control	After fermentation	Control	After fermentation
Calycosin-glu	286.5 ± 2.32^c^	47.86 ± 0.82^ef^	287.81 ± 1.64^c^	49.56 ± 0.83^de^	328.05 ± 1.57^b^	45.82 ± 0.96^f^	394.44 ± 0.42^a^	50.87 ± 1.19^d^	91.08 ± 0.40	32.72 ± 1.02
Genistin	8.07 ± 0.50^g^	28.77 ± 0.63^b^	11.33 ± 0.34^f^	22.21 ± 0.87^c^	13.76 ± 0.52^e^	35.38 ± 0.23^a^	16.54 ± 0.35^d^	34.52 ± 0.77^a^	165.98 ± 12.55	92.21 ± 0.94
Ononin	99.64 ± 1.23^c^	14.65 ± 0.70^f^	99.71 ± 1.88^c^	16.09 ± 0.82^ef^	108.88 ± 0.92^b^	17.06 ± 0.38^e^	119.22 ± 1.89^a^	19.49 ± 0.65^d^	82.43 ± 0.47	35.55 ± 1.69
Calycosin	41.08 ± 0.66^g^	62.34 ± 1.13^e^	44.23 ± 1.80^f^	77.11 ± 1.63^d^	65.00 ± 0.82^e^	157.71 ± 2.40^b^	82.75 ± 2.28^c^	207.32 ± 2.45^a^	53.37 ± 1.63	274.42 ± 1.56
Formononetin	23.88 ± 0.73^e^	24.73 ± 0.81^e^	23.97 ± 0.61^e^	16.76 ± 0.64^f^	37.63 ± 0.43^d^	40.19 ± 0.50^c^	54.19 ± 0.43^b^	56.76 ± 1.39^a^	90.5 ± 1.48	260.74 ± 7.97
AG IV	79.29 ± 0.84^g^	1581.3 ± 1.24^c^	79.78 ± 0.24^g^	1258.96 ± 2.12^d^	144.35 ± 1.14^f^	1651.78 ± 2.92^b^	270.41 ± 1.41^e^	2331.46 ± 1.86^a^	70.29 ± 0.64	117.37 ± 0.18
AG II	117.84 ± 2.57^g^	587.53 ± 0.86^c^	118.81 ± 0.43^g^	567.54 ± 2.88^d^	201.61 ± 0.59^f^	728.86 ± 2.34^b^	245.07 ± 1.68^e^	788.31 ± 2.47^a^	45.3 ± 0.24	85.37 ± 3.00
Total saponins	16.28 ± 0.97^e^	12.59 ± 0.38^f^	16.92 ± 0.79^e^	16.21 ± 0.72^e^	132.79 ± 2.36^c^	126.87 ± 2.53^d^	140.07 ± 0.86^a^	136.12 ± 1.86^b^	245.86 ± 4.96	437.95 ± 9.81
Total flavonoids	0.60 ± 0.06^e^	1.42 ± 0.22^cd^	0.97 ± 0.09^de^	1.24 ± 0.38^d^	1.72 ± 0.24^c^	3.16 ± 0.28^a^	2.25 ± 0.12^b^	3.37 ± 0.42^a^	98.37 ± 15.06	125.69 ± 19.86

Values are expressed as mean ± standard error from three replications. Means with different superscripts in the same row show significant difference (P < 0.05). Unit of calycosin-glu, genistin, ononin, calycosin, formononetin, AG IV and AG II here is μg/g. Unit of total saponins and total flavonoids here is mg/g.

In OP and GP, the contents of flavonoids and saponins of control and FRA had little change. After undergoing successive digestion in ENTI and ENTII, the contents of flavonoids and saponins increased, especially the total saponins increased significantly. The reason might be due to the fact that flavonoid and saponin components were in an acidic environment (during digestion in the stomach), which could suppress the release of them, while digestive enzymes in the intestine could hydrolyze proteins to release the protein-encapsulated flavonoids or saponins and they can exist stably in the weakly acidic environment^15^.

Bioaccessibility data of FRA and control are displayed in Table 4. The bioaccessibility of calycosin, formononetin, AG IV, AG II, total saponins and total flavonoids in the FRA were 274.34%, 260.61%, 117.36%, 83.63%, 437.83% and 125.28% and those of the control were 53.37%, 90.48%, 70.29%, 45.30%, 245.82% and 96.98%, respectively. The bioaccessibility of these components in the FRA were higher than those of the control. However, the bioaccessibility of calycosin-glu, genistin and ononin in the FRA were lower than those of the control. These results are possibly due to the glucosides have lots of oxhydryl, which are tightly bound to cellulose. After fermentation, the *Poria cocos* produced enzymes to hydrolyze cellulose, which can release the glucosides. And the multienzyme system produced by *Poria cocos* could promote the biotransformation of glucosides. On the other hand, the digestive enzymes could convert glucosides to aglycones during the in vitro digestion^16^. Thus the bioaccessibility of aglycones in the FRA were higher than those of control and the bioaccessibility of glucosides in the FRA were lower than those of control.

It is an interesting phenomenon that the contents of some bioactive components of control and FRA in fermentation experiment were higher than those in the vitro digestion experiment. For example, the content of calycosin in the control was 155.05 μg/g in Table 3 and 41.08 μg/g in Table 4 of OP. The content of calycosin in FRA was 75.57 μg/g in Table 3 and 62.34 μg/g in Table 4 of OP. On the one hand, it may be due to the solubility of the bioactive components in ethanol and digestion solution was different. On the other hand, the content of calycosin in FRA was higher than that of control in OP because the *Poria cocos* solid fermented *Radix Astragali* could promote the release of bioactive components. From this it can be seen that the *Poria cocos* solid fermented *Radix Astragali* could improve the bioaccessibility of *Radix Astragali*. In brief, in vitro digestion experiments validates that the FRA has higher bioaccessibility than control, which provides valuable information for FRA at different digestion stages.

### SEM analysis

**Figure 2:**
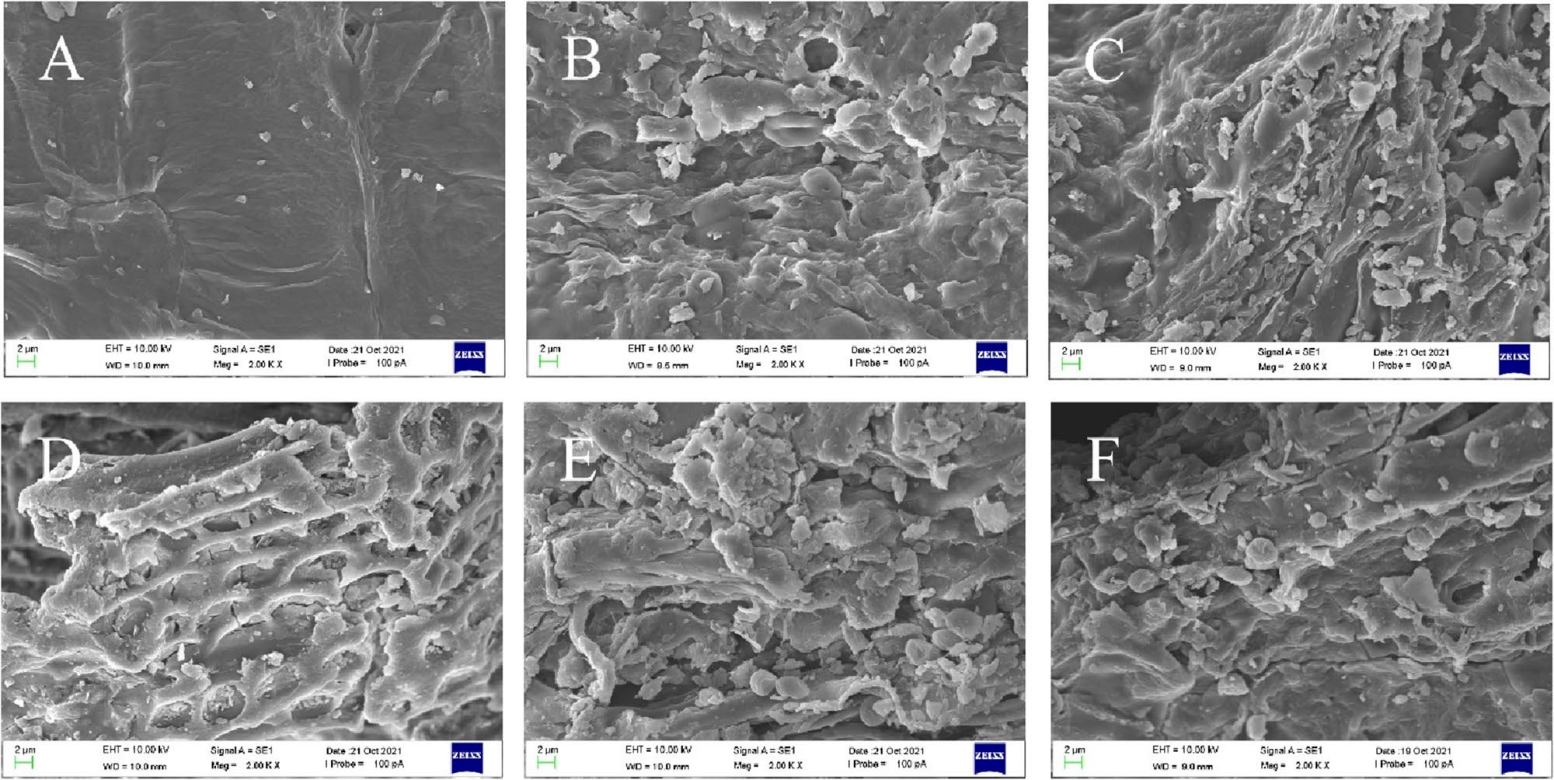
Scanning electron micrographs of each sample of Radix Astragali ((**A**) the control, (**B**) the control in the gastric digestion, (**C**) the control in the enteric II digestion, (**D**) the FRA, (**E**) the FRA in the gastric digestion, (**F**) the FRA in the enteric II digestion).

The samples were examined by EVO LS15 SEM at a magnification of 2000x, in order to study the structural alterations of the *Radix Astragali* by fermentation and in vitro digestion. Figure 2 shows the control (A), the control in the gastric digestion (B), the control in the enteric II digestion (C), the FRA (D), the FRA in the gastric digestion (E), and the FRA in the enteric II digestion (F). The surface of control was smooth, basically no holes and with a small amount of particulate matter attached. Figure 2A indicated that the cells of control were not obviously destroyed and still retained the intact tissue structure. After fermentation, the microstructures were disorganized in Fig. 2D. The surface of FRA residues was rough, porous and reticular structure. These results may be due to *Poria cocos* mycelium could directly enter the drug residue and destroy the internal cells of *Radix Astragali*.

After gastric digestion, the electron micrographs of both samples changed significantly. Under the action of pepsin and acid digestion fluid, the sample was severely damaged. The surface became uneven and many particles were observed. As can be seen from Fig. 2B, E the fermentation samples were destroyed more seriously than the control samples. The results may be related to the destruction of *Radix Astragali* by *Poria cocos*. After fermentation, the surface of *Radix Astragali* showed a porous reticular structure, which increased the exposure area and made the active ingredients easier to digest and dissolve. After enteric II digestion, both samples were strongly destroyed, and the electron micrographs of Fig. 2C,F were not significantly different from those of Fig. 2B,E.

### Antioxidant activity

The various components have antioxidant activity in *Radix Astragali*. In the fermentation and in vitro digestion, the contents of AG IV, AG II, calycosin and genistin showed significant changes, thus their standards were selected as the reference for the antioxidant experiments of *Radix Astragali*. Here, the antioxidant capacities of ascorbic acid (Vc), non-fermented *Radix Astragali* extracts (control), FRA extracts and their in vitro digestive products were assessed with a DPPH radical scavenging assay and ABTS radical scavenging assay.

**Figure 3:**
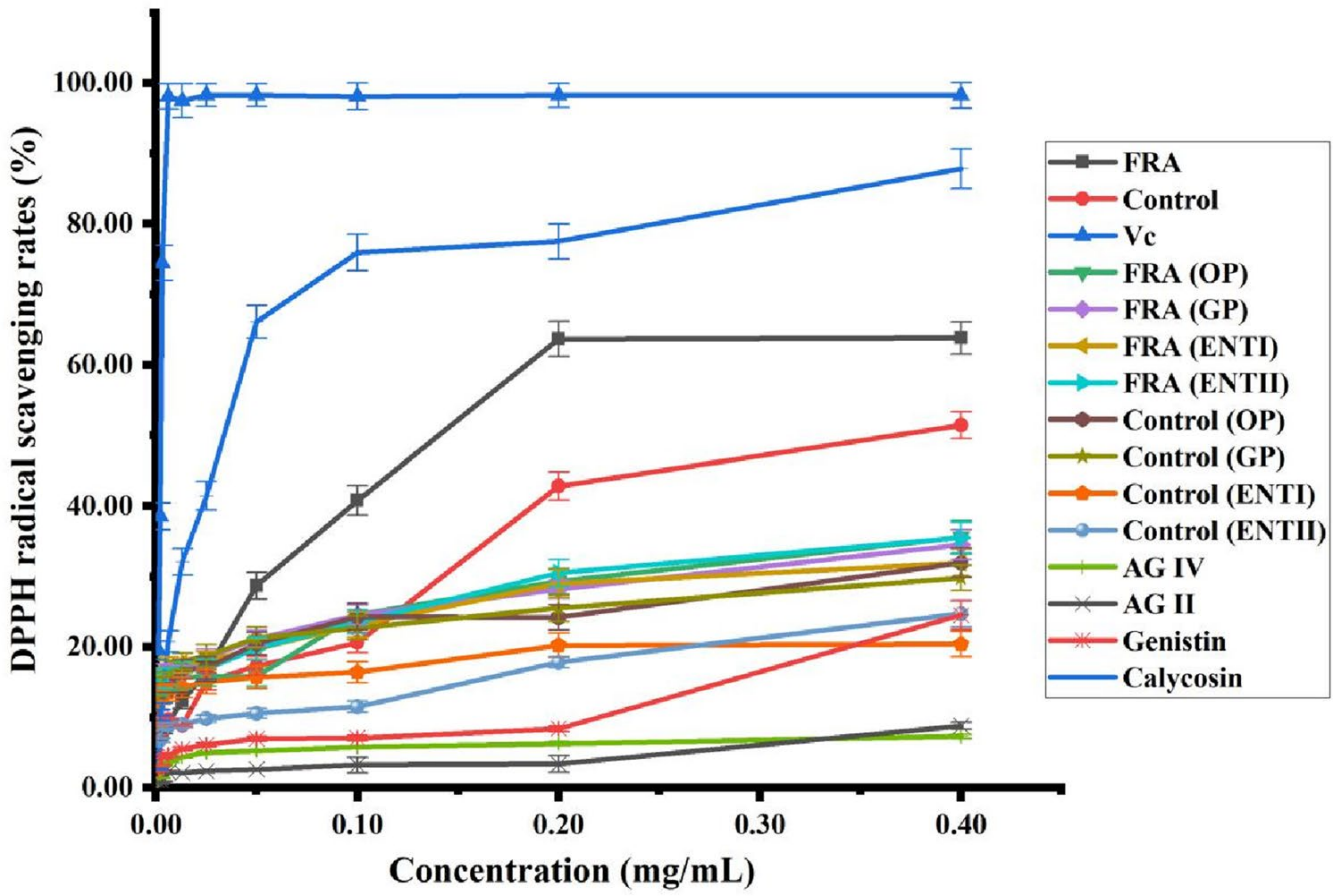
DPPH radical scavenging rate of each sample of Radix Astragali, standards and Vc.

The DPPH antioxidant results of extracts and in vitro digested samples of *Radix Astragali* are shown in Fig. 3. The IC_50_ value of FRA was 0.140 mg/mL, which was higher than the IC_50_ value of the reference Vc (0.002 mg/mL) and calycosin (0.034 mg/mL), but superior to that of control (0.367 mg/mL). At 0.4 mg/mL, the DPPH free radical scavenging rate of the sample from high to low were: Vc 98.25%, calycosin 87.86%, FRA 63.81%, control 51.43%, FRA (OP) 35.59%, FRA (ENTII) 35.50%, FRA (GP) 34.52%, control (OP) 31.96%, FRA (ENTI) 31.94%, control (GP) 29.81%, genistin 24.52%, control (ENTII) 24.72%, control (ENTI) 20.42%, AGII 8.81% and AG IV 7.30%.

**Figure 4:**
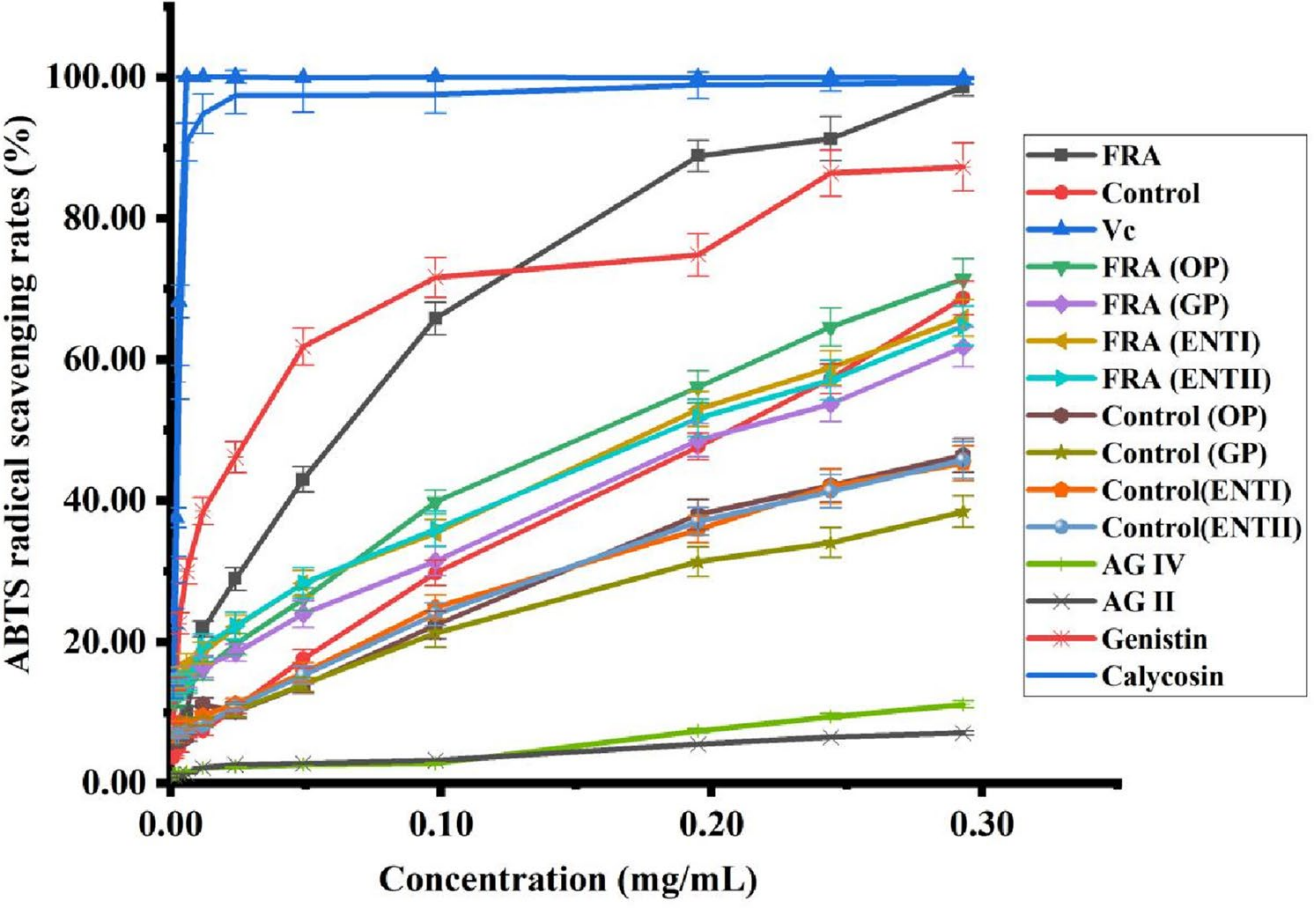
ABTS radical scavenging rate of each sample of Radix Astragali, standards and Vc.

The ABTS antioxidant results of extracts and in vitro digested samples of *Radix Astragali* are shown in Fig. 4. The IC_50_ value of FRA extracts was 0.064 mg/mL, which was higher than the IC_50_ value of the reference Vc (0.002 mg/mL), calycosin (0.003 mg/mL) and genistin (0.030 mg/mL) but superior to that of control (0.207 mg/mL). In the range of 0.01–0.30 mg/mL, the antioxidant capacity of the sample increased with the increase of mass concentration. At 0.29 mg/mL, the ABTS free radical scavenging rate of the sample from high to low were: Vc 99.86%, calycosin 99.28%, FRA 98.60%, genistin 87.26%, FRA (OP) 71.39%, control 68.72%, FRA (ENTI) 65.86%, FRA (ENTII) 64.73%, FRA (GP) 61.76%, control (OP) 46.44%, control (ENTII) 45.73%, control (ENTI) 45.30%, control (GP) 38.46%, AG II 11.14% and AG IV 7.09%.

After fermentation and in vitro digestion, the DPPH and ABTS antioxidant capacities of FRA were all higher than that of control. However, the antioxidant activity of FRA and control decreased after in vitro digestion, which may be due to the weight of the extractum from the digestive fluid was higher than that of the FRA and control. Under the same dry mass concentration, the contents of antioxidant components in the antioxidant samples decreased, which affected the antioxidant capacity.

## Conclusions

In conclusion, a scheme of solid fermentation of *Radix Astragali* with *Poria cocos* was proposed to increase the contents of the main active compound in *Radix Astragali* (AG IV). By optimizing the fermentation process, the best fermentation conditions are obtained: inoculation amount 8 mL; fermentation time 10 days; fermentation humidity 90%. Under optimal conditions, the content of AG IV increased from 384.73 to 1986.49 μg/g by 5.16-fold. Moreover, the bioaccessibility of FRA was investigated by in vitro digestion. After in vitro digestion, the contents of genistin, calycosin, formononetin, AG IV, AG II and total flavonoids of FRA in ENTII were 34.52 μg/g, 207.32 μg/g, 56.76 μg/g, 2331.46 μg/g, 788.31 μg/g, 3.37 mg/g, which were 2.08-fold, 2.51-fold, 1.05-fold, 8.62-fold, 3.22-fold and 1.50-fold higher than those of the control, respectively. And the bioaccessibility of calycosin, formononetin, AG IV, AG II, total saponins and total flavonoids of the FRA were 274.34%, 260.61%, 117.36%, 83.63%, 437.83% and 125.28% and those of the control were 53.37%, 90.48%, 70.29%, 45.30%, 245.82% and 96.98%, respectively. As observed through SEM, the surface of FRA and its in vitro digestion residue was rough and porous, appeared clearer reticular structure, and the surface area increased than control. In the DPPH and ABTS radical scavenging assay, the IC_50_ value of FRA was 0.140 mg/mL and 0.064 mg/mL, which were superior to that of control (0.367 mg/mL, 0.207 mg/mL). These results showed that the antioxidant of FRA was higher than that of control. After in vitro digestion, the FRA still have good antioxidant activity. In this study, *Poria cocos* was used to ferment *Radix Astragali* for the first time to complete the biotransformation of bioactive components. The FRA has high bioavailability and strong antioxidant activity, which provided a basis for the development and utilization of *Radix Astragali*.

## Methods

### Materials and reagents

*Radix Astragali*, the dried root of *Astragalus membranaceus* (Fisch.) Bge. var. *mongholicus* (Bge.) Hsiao was purchased from Shanxi Hunyuan Wansheng Huangqi Development Co., Ltd (Shanxi, China) and authenticated by Prof. Ying Lin at Binzhou Medical University (Yantai, Shandong). Voucher specimens were deposited in the herbarium of the Experimental Center of Chinese Medicine of Binzhou Medical University and the accession number was 2021–1003. *Radix astragali* was pulverized by a 800A disintegrator (Yongkang Hardware and Medical Instrument Plant, China) and passed through mesh sieve to obtain a fine powder with a particle size of 600 μm. *Poria coc*os (bio-08656) was purchased from Beijing Bai Ou Bo Wei Biotechnology Co., Ltd. (Beijing, China). Formic acid, methanol and acetonitrile of chromatographic grade were purchased from Tianjin Kemiou Chemical Reagent Co., Ltd. (Tianjin, China). Ultrapure water used in the whole experiment was purified by a Milli-Q water purification system from Millipore (Bedford, MA, USA). PDA medium and potato liquid medium were purchased from Qingdao Haibo Biotechnology Co., Ltd. (Qingdao, China). Potassium phosphate, potassium dihydrogen phosphate, sodium carbonate, potassium persulfate and sodium hydroxide were purchased from Sinopharm Chemical Reagent Co., Ltd. (Shanghai, China). Salivary amylase (BR, 4000u/g), pepsin (USP grade, 1:3000), gastric lipase (BR, 30,000u/g), pancreatic enzyme (BR, 1600u/g) and pig bile powder (BR) were purchased from Shanghai Yuanye biotech Co. (Shanghai, China). 1,1-diphenyl-2-picrylhydrazyl and 2,2-hydrazine-bis(3- ethylbenzothiazoline-6-sulfonic acid) diamine salt were purchased from Shanghai Macklin Biochemical Technology Co., Ltd. (Shanghai, China). Sodium nitrite was purchased from Tianjin Bodi Chemical Co., Ltd. (Tianjin, China). Aluminum nitrate was purchased from Shanghai walkai Biotechnology Co., Ltd. (Shanghai, China), Anhydrous ethanol was purchased from Tianjin Yongda Chemical Reagent Co., Ltd. (Tianjin, China). Vanillin was purchased from Tianjin Guangfu Fine Chemical Research Institute. Rutin (≥ 98%), Ononin (≥ 98%), Genistin (≥ 98%), Calycosin (≥ 98%), Calycosin-glu (≥ 99%), Formononetin (≥ 99%), AG II (≥ 98%) and AG IV (≥ 98%) were purchased from Chengdu Pusi biotech Co. (Chengdu, China).

### Statement for this study

All methods in this study were carried out in accordance with the relevant guidelines and regulations of the Pharmacopoeia of the People's Republic of China.

### Fermentation

#### Growth curve of *Poria cocos* and preparation of seed fermentation broth

*Poria cocos* was inoculated on the culture dish with 46.0 g/L PDA medium, until the mycelium spread all over the culture dish. Then, the inoculated mycelium was cultured in 100 mL of 26.1 g/L potato liquid medium on a ZQPW-70 shaker incubator (Tianjin Laboratory Instrument Equipment Co., Ltd, Tianjin, China) at 27 °C, 180 rpm for 2 days–10 days. Following incubation, the mycelia were washed with water during filtration. Samples were placed in an oven at 60 ℃ until constant weight, and then the dry weight of the mycelia was measured. Each group had three parallel samples.

#### Optimization of solid-state fermentation conditions

**Figure 5:**
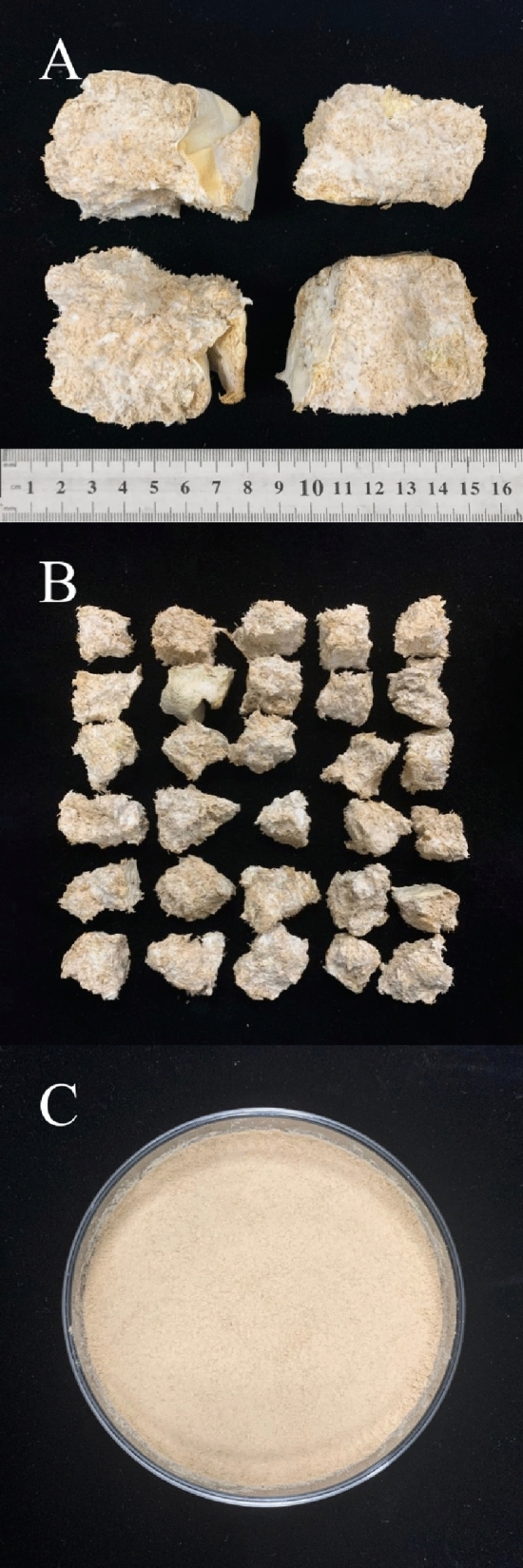
Photo of *Poria cocos* fermented Radix Astragali ((**A**) the fermented *Radix Astragali*, (**B**) the small pieces of FRA, (**C**) the powder of FRA).

Twenty grams of *Radix Astragali* powder and 5 mL-25 mL ultrapure water were added to the fermentation bags. The mixture was put in an YXQ-Sll autoclave (Shanghai Boxun Medical Biological Instrument Co., Ltd, Shanghai, China) at 121 °C for 25 min. After that, 4 mL–9 mL of seed fermentation broth was inoculated on the medicinal powder of fermenting bag. Then, the mixture was incubated in a GSP-9160MBE constant temperature and humidity incubator (Shanghai Boxun Medical Biological Instrument) at 27 °C for 6 days–11 days. Each group had three parallel samples. The photo of product of *Poria cocos* fermented *Radix Astragali* was shown in Fig. 5A. Control experiments were *Radix Astragali* powder only with autoclaved treatment without *Poria cocos.* And ultrapure water replaced the seed fermentation broth to maintain the same moisture level.

#### In vitro digestion process

The in vitro simulated gastrointestinal method consists of four phases: oral phase OP, GP, ENTI and ENTII.

#### Sample preparation

The FRA (Fig. 5A) was broken into small pieces (Fig. 5B) and dried at 60 °C. The dried samples were grounded into powders using a disintegrator and were passed through a 60-mesh sieve (Fig. 5C).

#### Simulated oral digestion

Five grams of the fermented sample and control were added into different conical bottle after sterilization. Ten milliliter 20 m mol/L phosphoric acid buffer was added, 0.9% normal saline was added to 125 mL, pH value was adjusted to 7.0 with 1 mol/L NaOH solution and kept at 37 °C for 10 min. Finally, 0.2 mg salivary amylase was added, mixed evenly. And put into a constant temperature water bath oscillator preheated at 37 °C, 55 rpm for 2 min in the dark. Four groups were digested at the same time. After digestion, they were heated to boiling in water for 2 min. One group was stored and the remaining 3 groups were continued for the next stage of digestion. Three parallel samples for each group.

#### Simulated gastric digestion

After oral digestion, the remaining three groups of digestion samples were added to 125 mL with 0.9% normal saline. The pH value was adjusted to 2.0 with 1 mol/L HCl solution after mixing, and kept for 10 min at 37 °C in constant temperature water bath oscillator. Then, 0.375 g pepsin and 0.1125 mg lipase were added and mixed well. And put into a constant temperature water bath oscillator preheated at 37 °C, 150 rpm, for 2 h in the dark. After digestion, they were heated to boiling in water for 2 min. One group was stored and the remaining two groups were continued for the next stage of digestion.

#### Simulated enteric digestion

Referring to the research of Ningtyas, et al.^14^, the enteric digestion phase was divided into two phases: ENTI and ENTII.

After gastric digestion, the remaining two groups of digestion samples were added to 125 mL with 0.9% normal saline. The pH value was adjusted to 4.7 with 1 mol/L NaOH solution, and the sample was kept for 10 min at 37 °C. Then, 0.125 g trypsin and 1.25 g bile were added and mixed well. The enteric I stage was digested for 2 h at 37 °C for 150 rpm in a water bath oscillator in the dark condition. After digestion, they were heated to boiling in water for 2 min. One group was stored and the other was continued for the next stage of digestion.

The last group of digested sample was added to 125 mL by the same way, the pH was adjusted to 6.5 with 1 mol/L NaOH solution, and it was kept for 10 min at 37 °C. Then, added 0.125 g trypsin and 1.25 g bile, mixed well. The enteric II stage was digested for 2 h at 37 °C for 150 rpm in a water bath oscillator in the dark condition. After digestion, it was heated to boiling in water for 2 min and stored.

The stored samples of each digestion stage were centrifuged at 12,000 rpm for 5 min at 4 °C. The supernatant was collected and used to determine chemical composition. The rest residue was dried at 60 °C and used for SEM.

#### Bioaccessibility calculations

Bioaccessibility was considered as the concentration of bioactive compounds released from the food matrix by in vitro enteric digestion. The bioaccessibility is calculated according to the following equation:

1 $${\text{Bioaccessibility}}\% = [{\text{BC}}_{{{\text{digested}}}} /{\text{BC}}_{{{\text{non}} - {\text{digested}}}} ] \times {1}00\%$$

where BC_digested_ and BC_non-digested_ correspond to the bioactive compound concentration in digested and non-digested *Radix Astragali*, respectively.

### Component determination

#### Sample preparation

Five g sample was weighted accurately and 100 mL of 70% ethanol was added to a conical flask. The mixture was extracted by ultrasound bath for 60 min with the power of 100 W. The extracted samples were filtered and centrifuged at 12,000 rpm for 5 min at 4 °C. The supernatant was collected and used to determine chemical composition.

#### Determination of total flavonoids

The total flavonoid content was determined following methods by Liu, et al.^17^, and slightly modified. Rutin was used as a standard for the calibration curve. Rutin was dissolved in ethanol with an initial concentration of 0.2 mg/mL. The different volumes of the stock solution with 0, 0.2, 0.4, 0.8, 1.2, 1.6, 2.0 and 2.4 mL were transferred into 10 mL volumetric flasks and were added 2 mL 70% ethanol. Shaken evenly, 0.3 mL of 5% NaNO_2_ solution was added and hold for 6 min. Then, 0.3 mL of 10% AlCl_3_ solution was added and the mixture was shaken evenly and hold for 6 min. After that, 4 mL of the 4% NaOH solution was added in the volumetric flask. The mixture was fixed volume to scale with 70% ethanol and hold for 15 min. The absorbance of these mixtures was measured at 510 nm using TU-1810 APC UV–Visible spectrophotometer (Beijing Persee General Instrument Co., Ltd, Beijing, China) and the standard curve was established.

Three millilitre of sample solution prepared above were transferred in 10 mL volumetric flasks. The absorbency of the sample was determined by the colorimetric method as described above.

#### Determination of total triterpenoid saponins

The total saponins content was determined following methods by^18^, and slightly modified. AG IV was used as a standard for the calibration curve. AG IV was dissolved in ethanol with an initial concentration of 0.5 mg/mL. The different volumes of the stock solution with 0.00, 0.05, 0.10, 0.20 0.30, 0.40 mL were transferred into 10 mL volumetric flasks and were added 70% ethanol up to 0.5 mL. Shaken evenly, 0.5 mL of 8% vanillin ethanol solution and 5.0 mL of 72% sulfuric acid were added. After mixing evenly, the reaction mixture was incubated at 62 ℃ for 20 min. Then, the mixture was cooled in an ice water bath to room temperature. The absorbance of these mixtures was measured at 540 nm using TU-1810 APC UV–Visible spectrophotometer and the standard curve was established.

Sample solution (0.5 mL) prepared above were transferred in 10 mL volumetric flasks. The absorbency of the sample was determined by the colorimetric method as described above.

#### High performance liquid chromatography (HPLC) method

Astragalus flavonoids were analyzed by HPLC, using a Thermo Scientific Dionex Ultimate 3000 series HPLC System (Dionex, USA), diode array detector (Dionex, USA) and a Kromasil 100–5-C18 column (4.6 by 250 mm). Column temperature was maintained at 30 °C with 10 µL injection volume. The mobile phase consisted of 0.05% formic acid in water (A) and acetonitrile (B) using the following gradient elution sequence: 0–5 min, 0–5% B; 5–10 min, 5–20% B; 10–25 min, 20–25% B; 25–35 min, 25–30% B; 35–40 min, 30–35% B; 40–54 min, 35–40% B; 54–55 min, 40–0% B. The flow rate was set at 1 mL/min. Flavonoids were detected and quantified by monitoring the absorbance at 254 nm, including calycosin-glu, genistin, Ononin, calycosin and formononetin, their molecular formulae and chemical structures were shown in Table 5. The concentrations of different analytes were determined according to the corresponding standard curves. The regression equations were y_1_ = 640.7x−0.1856 (R^2^ = 0.9994, n = 6); y_2_ = 886.38x−0.2805 (R^2^ = 0.9995, n = 6); y_3_ = 746.76x−0.2932 (R^2^ = 0.9995, n = 6); y_4_ = 995.48x−0.383 (R^2^ = 0.9995, n = 6); y_5_ = 1234x−0.5045 (R^2^ = 0.9994, n = 6), where y was the peak area of analyte, and x was the concentration of analyte (mg/mL).

**Table 5:** Chemical structures of relevant flavonoids and saponins in *Radix Astragali*. Glu, glucose; Ac, acetyl group.

Number	Name of compounds	Molecular formula	Structure	R
1	Calycosin glucoside	C_22_H_22_O_10_		R_1_ = H, R_2_ = OH + Glu, R_3_ = OH, R_4_ = OCH_3_
2	Genistin	C_21_H_20_O_10_		R_1_ = OH, R_2_ = OH + Glu, R_3_ = H, R_4_ = OH
3	Ononin	C_22_H_22_O_9_		R_1_ = H, R_2_ = OH + Glu, R_3_ = H, R_4_ = OCH_3_
4	Calycosin	C_16_H_12_O_5_		R_1_ = H, R_2_ = OH, R_3_ = OH, R_4_ = OCH_3_
5	Formononetin	C_16_H_12_O_4_		R_1_ = H, R_2_ = OH, R_3_ = H, R_4_ = OCH_3_
6	AG II	C_43_H_70_O_15_		R_1_ = Ac, R_2_ = H, R_3_ = H, R_4_ = Glu
7	AG IV	C_41_H_68_O_14_		R_1_ = H, R_2_ = H, R_3_ = H, R_4_ = Glu

A DGU-20A3R HPLC system (SHIMADZU, Japan) coupled with an ELSD-16 (SHIMADZU, Japan) was used for the determination of the AG IV and AG II, whose molecular formulae and chemical structures were shown in Table 5. The chromatographic separations were performed on a Phenomenex C18 column (4.60 × 250 mm, 5 μm) maintained at room temperature. The mobile phases consisted of acetonitrile (A) and water (B) with a gradient elution as follows: 0–20 min, 96–80% B; 20–40 min, 80–70% B; 40–65 min, 70–40% B; 65–70 min, 40–96% B; 70–80 min, 96% B. A constant flow rate of 1.0 mL/min was employed. The drift tube temperature of the ELSD was set at 100 °C, and the carrier gas flow rate was 2.5 L/min. The sample injection volume was 20 μL. The regression equations for AG IV and AG II were y_6_ = 1.5346x + 0.3752 (R^2^ = 0.9980, n = 6); y_7_ = 1.644x + 0.1816 (R^2^ = 1.0000, n = 6), where y was the peak area of analyte, and x was the concentration of analyte (mg/mL).

#### Scanning electron microscopy (SEM)

A EVO LS15 scanning electron microscope (Zeiss, Germany) with Q150RS ion sputtering coater (Quorum, UK) were used to examine the morphological alterations of samples. The samples of before and after fermentation and the sample residue from each stage of in vitro digestion were dried at 60 °C and crushed. The crushed samples were fixed on the SEM sample stage with conductive adhesive and were coated with gold on the sample surface using an ion sputter coater. Then, the samples were observed at an accelerating voltage of 10 kv (2000× magnification).

### Antioxidant activity

#### Sample preparation

The supernatant prepared above were concentrated to dryness in RE-52AA rotary evaporator device (Shanghai Yarong Biochemical Instrument Factory, Shanghai, China) under vacuum at 55 °C. The dried extract was dissolved by anhydrous ethanol to prepared different concentrations sample solution for the determination of antioxidant activity.

#### DPPH radical scavenging assay

The antioxidant activity was performed through 2,2-diphenyl-1-picrylhydrazyl (DPPH) free-radical scavenging activity. The antioxidant activity was determined according to the method of Amarowicz, et al.^19^ and Chen, et al.^20^, with some modification. One mg/mL sample solution 100 μL was mixed with 1 mL of 0.08 mg/mL DPPH ethanol solution. Then, anhydrous ethanol was added to 4 mL. The mixture was shaken vigorously and incubated in darkness for 30 min. The absorbance of the solution was measured at 517 nm using TU-1810 APC UV–Visible spectrophotometer. Vc was used as a reference compound. All tests were carried out in triplicate. The free radical scavenging activity is calculated according to the following equation:

2 $$\mathrm{DPPH\%}=[\mathrm{AB}-\mathrm{AA}] /\mathrm{AB}\times 100\mathrm{\%}$$

where AB is the absorbance of the control (DPPH without sample), AA is the absorbance of the reaction mixture.

#### ABTS radical scavenging assay

The antioxidant activity was performed through 2,2-hydrazine bis (3-ethylbenzothiazoline-6-sulfonic acid) diamine salt radical cation (ABTS^+^) scavenging activity. The antioxidant activity was determined according to the method of Ali et al.^21^, with some modification. Four millilitre of ABTS solution was added to 0.1 mL of various concentrations of the sample solution. The mixture was shaken vigorously and incubated in darkness for 6 min. The absorbance of the solution was measured at 734 nm using TU-1810 APC UV–Visible spectrophotometer. Vc was used as reference compound. The free radical scavenging activity is calculated according to the following equation :

3 $${\text{ABTS}}^{ + } \% = [{\text{AB}} - {\text{AA}}]/{\text{AB}} \times {1}00\%$$

where AB is the absorbance of the control (ABTS without sample), AA is the absorbance of the reaction mixture.

### Statistical analysis

Statistics were done using SPSS 26 software. All the data were presented as mean ± standard error (SE) from three independent parallel experiments. Significant differences among the means of samples were analyzed by Duncan’s test with a 95% confidence level^14^. Origin 2018 software was used to graph the results.

## Data Availability

The datasets used and/or analysed during the current study are available from the first author Cai-Yun Chen on reasonable request.
